# Alarm Odor Compounds of the Brown Marmorated Stink Bug Exhibit Antibacterial Activity

**DOI:** 10.4172/2472-0992.1000119

**Published:** 2016-07-04

**Authors:** Steven Sagun, Elliot Collins, Caleb Martin, E Joseph Nolan, Joseph Horzempa

**Affiliations:** Department of Natural Sciences and Mathematics, West Liberty University, USA

**Keywords:** *Halyomorpha halys*, Brown marmorated stink bug, Anti-microbial, Anti-bacterial, Trans-2-octenal, Trans-2-decenal

## Abstract

Some insects release scented compounds as a defense against predators that also exhibit antimicrobial activity. Trans-2-octenal and trans-2-decenal are the major alarm aldehydes responsible for the scent of *Halyomorpha halys*, the brown marmorated stink bug. Previous research has shown these aldehydes are antifungal and produce an antipredatory effect, but have never been tested for antibacterial activity. We hypothesized that these compounds functioned similarly to the analogous multifunctional action of earwig compounds, so we tested whether these aldehydes could inhibit the growth of bacteria. Disk diffusion assays indicated that these aldehydes significantly inhibited the growth of Methicillin-resistant *Staphylococcus aureus*, *Escherichia coli, and Pseudomonas aeruginosa, in vitro*. Moreover, mealworm beetles (*Tenebrio molitor*) coated in stink bug aldehydes showed a substantial reduction in bacterial colonization compared to vehicle-treated insects. These results suggest that brown marmorated stinkbug aldehydes are indeed antibacterial agents and serve a multifunctional role for this insect. Therefore, stinkbug aldehydes may have potential for use as chemical antimicrobials.

## Introduction

The brown marmorated stink bug, *Halyomorpha halys*, is an invasive pest insect that was introduced to the United States in 1996 [1–4]. Glands of the ventral thorax and dorsal abdomen of this insect mediate the release of a pungent alarm signal that repels predators [5, 6].

The major components of the anti-predatory secretions of *Halyomorpha halys* are two aldehyde compounds: trans-2-octenal (OCT) and trans-2-decenal (DEC) [7, 8]. In addition to warding off predators, these compounds also exhibit anti-fungal activity indicating that they serve a protective role against entomophagous fungi [9].

Like stink bugs, earwigs also secrete multifunctional compounds. These insects release exudates containing benzoquinones [10]. These compounds provide the earwigs protection from predators as well as entomopathogenic fungi, bacteria, and nematodes [10]. We hypothesize that similarly to the earwig benzoquinones, stinkbug aldehydes are also multifunctional compounds that may possess anti-bacterial activity.

## Experimental Section

### Cultivation of bacteria

Brain Heart Infusion Broth (BHI) was prepared according to the instructions of the manufacturer (BD). This medium was inoculated with *Staphylococcus aureus* Rosenbach (ATCC BAA-1556, Methicillin-Resistant *S. aureus*, MRSA), *Escherichia coli* [West Liberty University Microbiology Culture Collection [11], or *Pseudomonas aeruginosa* 1244 [12] bacteria. Cultures were incubated at 37°C with agitation until they reached stationary phase.

### Disk diffusion assays

Disk diffusion assays measuring the antimicrobial effect of stink bug aldehydes were conducted in a similar manner as previously described [13]. Briefly, broth cultures were diluted to an optical density (A600) of 1.0. 0.1 ml of this cell suspension was spread plated onto solid medium (chocolate II agar; 10 cm diameter plate; 15 ml agar per plate). Sterile Whatman filter disks infused with 10 μL 8% (v/v in DMSO) trans-2-octenal (OCT), 40% OCT, 8% trans-2-decenal (DEC), 40% DEC, a mixture of OCT and DEC (4% or 20% v/v) or DMSO were placed onto the surface of these plates that were subsequently incubated at 37°C overnight. OCT (TCI America) and DEC (Acros Organics) were purchased from Fisher Scientific. The diameters of the zones of inhibition were measured using a metric ruler. Disk diffusion assays were conducted at least three times where similar results were observed in each experiment.

### *In vivo* beetle colonization assays

*Tenebrio molitor* beetles (Best Bet Inc.) were coated in OCT+DEC and the number of viable surface microbes was determined. To coat these beetles, 40 μL of a mixture of OCT and DEC (20% v/v each in DMSO) or DMSO was pipetted onto the dorsal surface. Beetles were placed in standard potting soil for 24 h at room temperature. Subsequently, visible soil was manually removed using sterile tools. Each beetle was placed in 2 ml sterile BHI broth and bacteria were liberated using a vortex (two 3 second bursts). Beetles were aseptically removed from BHI broth after vortexing. The liberated bacteria were serially diluted and plated to determine colony forming units (CFU). The *in vivo* beetle colonization assays were conducted in duplicate with similar results observed in each experiment. Student’s t tests for statistical comparison of beetle colonization were calculated using GraphPad Prism 6.0.

### Aldehyde structures

OCT and DEC structures were generated using ChemDraw by typing the chemical names of these compounds.

## Results and Discussion

**Figure 1:**
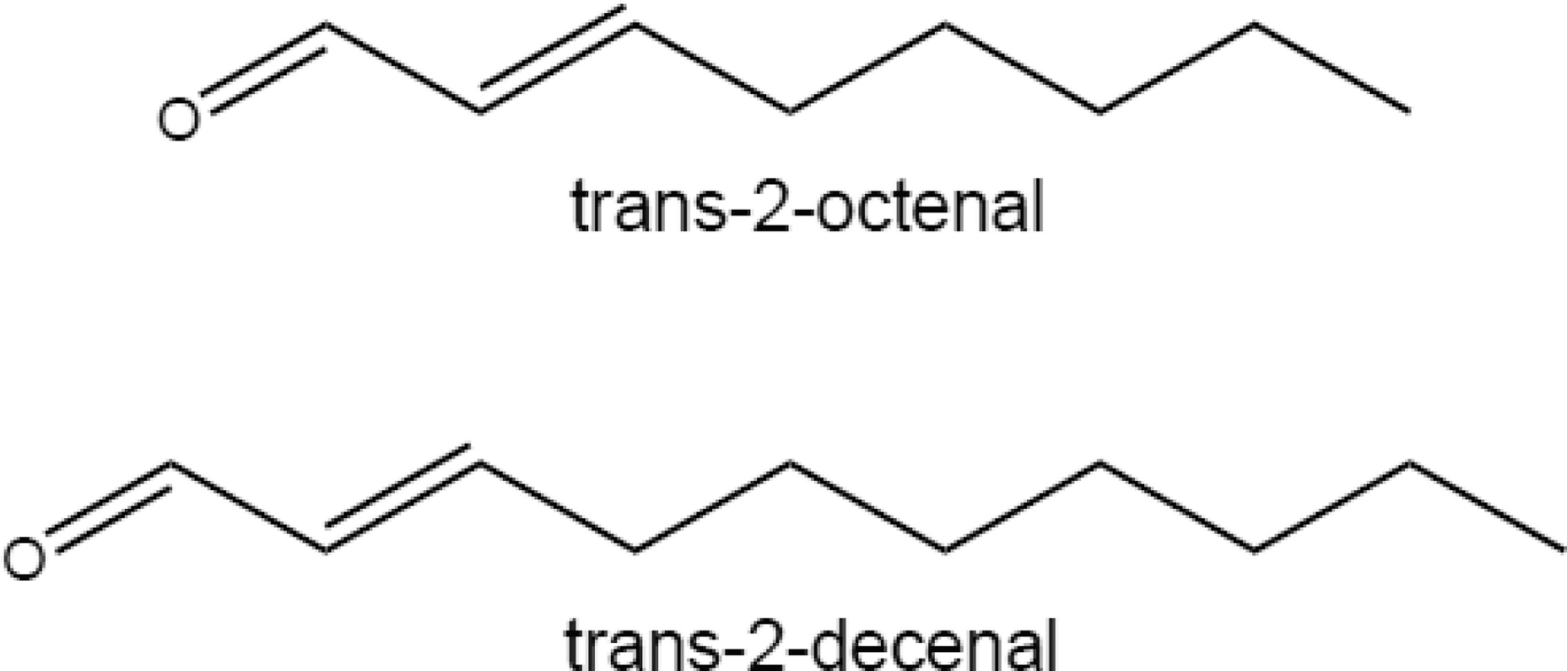
Stink bug aldehyde structures. OCT and DEC structures were generated using ChemDraw.

**Figure 2:**
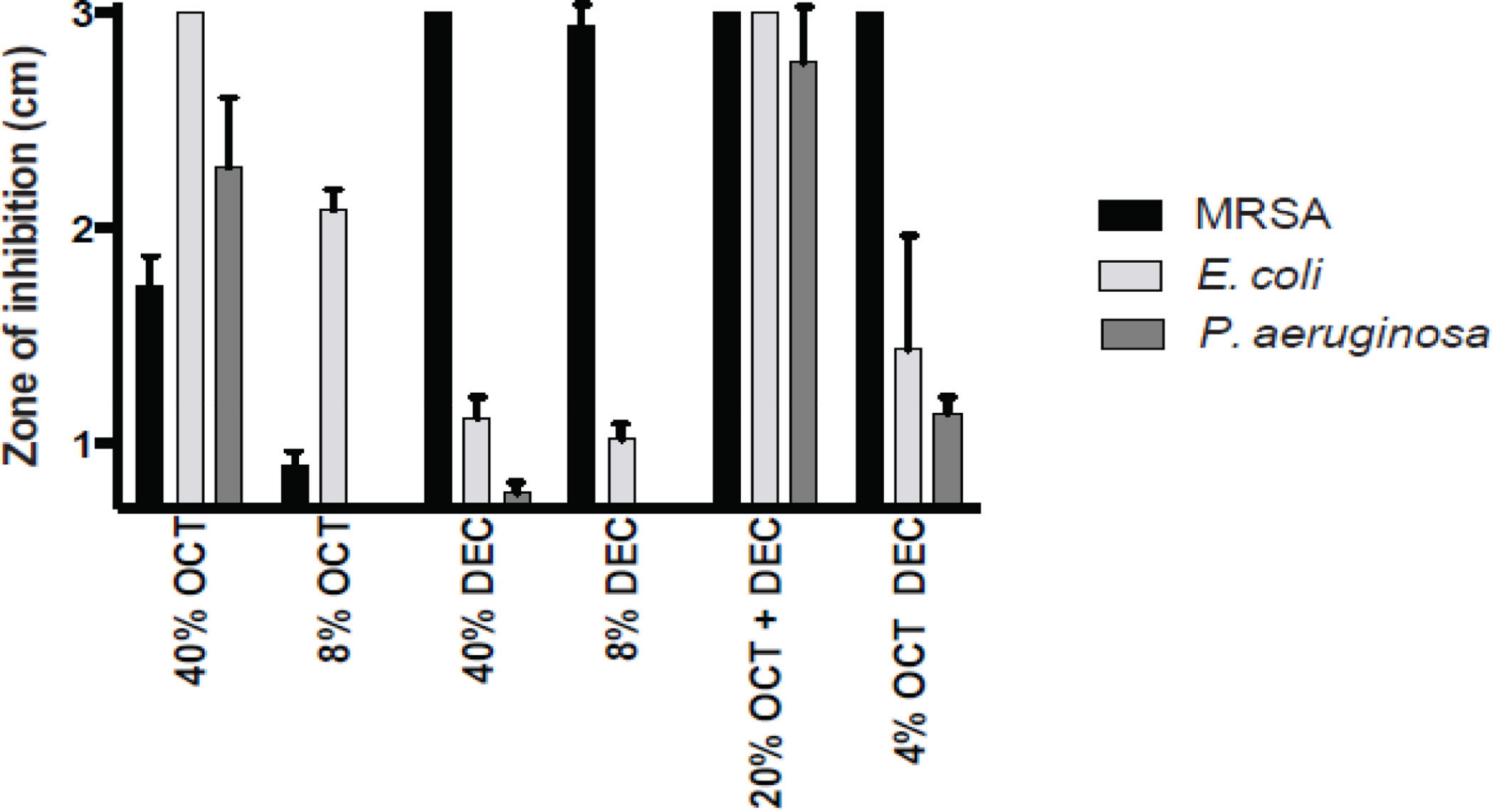
Disk diffusion assays on chocolate agar plates. Values were measured in centimeters and carried out in three replicates for each condition. DMSO was used as control. A representative of three separate experiments is shown.

Some insects release scented compounds as a defense against predators. Occasionally these compounds are multifunctional and exhibit antimicrobial effects as well as has been observed in three earwig species [10]. The brown marmorated stink bug (*Halyomorpha halys*) also secretes volatile compounds (OCT and DEC) to deter predators [6–8, 14] (Figure 1). The purpose of this study was to determine whether OCT and DEC exhibit antibacterial activity. Disk diffusion assays were conducted in which different concentrations and combinations of OCT and DEC were added to filter disks placed on solid growth medium that had been lawn-inoculated with *Staphylococcus aureus* MRSA, *Escherichia coli*, or *Pseudomonas aeruginosa* bacteria. Following incubation, zones of inhibition were measured. Both *Staphylococcus aureus* MRSA and *Escherichia coli* were significantly inhibited by OCT and DEC at either concentration that was used (Figure 2, P<0.001). However, only OCT at the highest concentration used (40%) was able to significantly inhibit the growth of *Pseudomonas aeruginosa* (Figure 2, P<0.0001). To determine whether the combination of these two aldehydes enhances their antimicrobial activity, disk diffusion assays were conducted in which both OCT and DEC were added to the filter disks at the concentration indicated. The combination of OCT and DEC at the lowest concentration tested (4%) significantly inhibited the growth of all three bacteria tested, including *Pseudomonas aeruginosa* (Figure 2)*.* Increasing the concentration to 40% completely inhibited the growth of all three microorganisms (Figure 2). This indicates that the anti-predatory aldehyde compounds (OCT and DEC) of *Halyomorpha halys* exhibit anti-bacterial properties, and the combined presence of these molecules enhances their anti-microbial efficacy.

**Figure 3:**
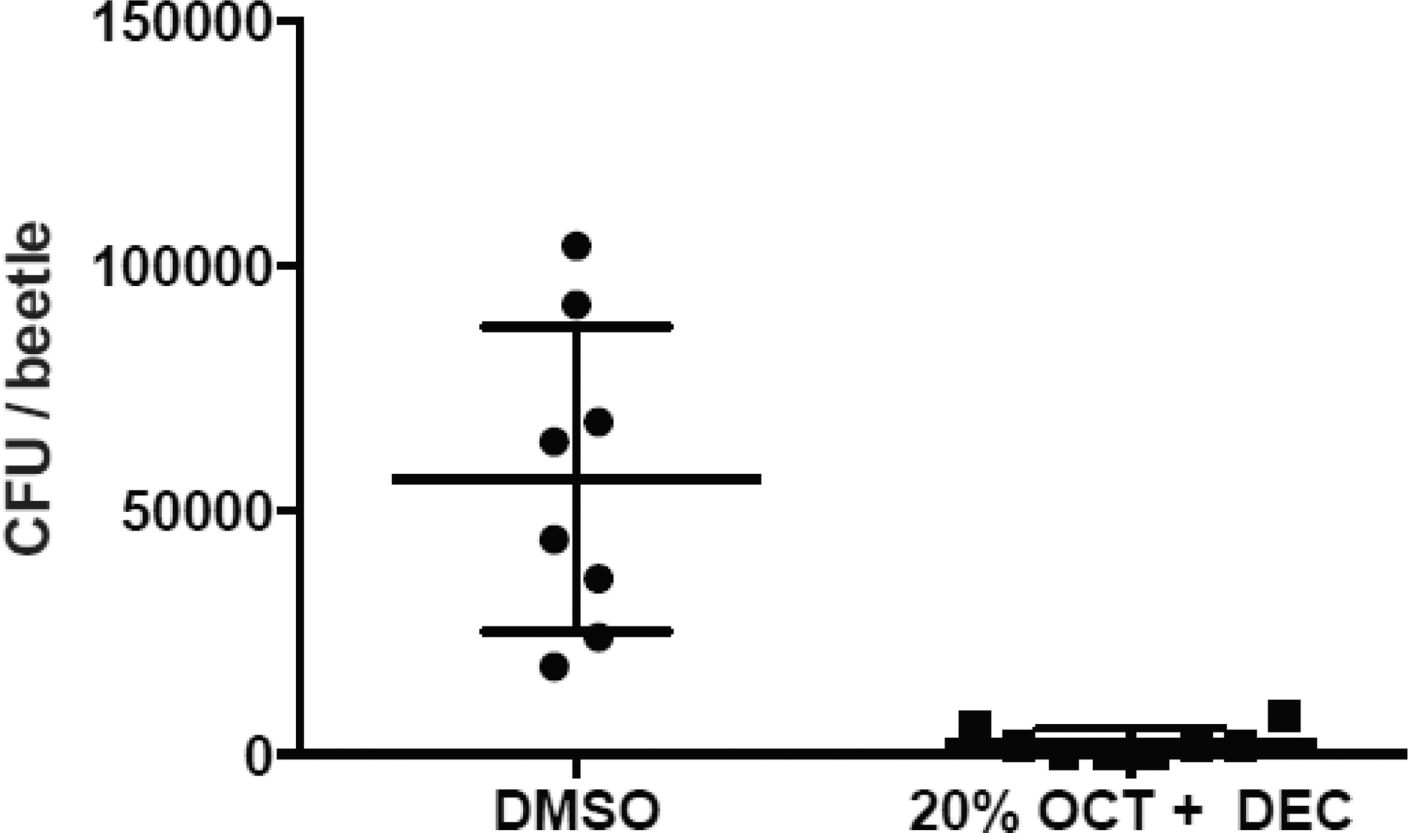
Stink bug aldehydes inhibit bacterial colonization of mealworm beetles. *Tenebrio molitor* beetles were coated in OCT+DEC and the number of viable surface microbes was determined. Treatment with stink bug aldehydes showed significant inhibition of bacterial colonization on the exoskeleton mealworm beetles vs. DMSO control. Individual data points are plotted with mean ± SD. A representative of duplicate experiments is shown. Data were analyzed using a student’s t-test, ^***^P<0.001.

Because OCT and DEC exhibited antimicrobial activity *in vitro* we wanted to further examine whether these compounds could prevent colonization of bacteria on an insect. To test this, 0.04 ml of 20% OCT and DEC (or DMSO as a control) was added to adult mealworm beetles (*Tenebrio molitor*) which were placed in soil for 24 hours at room temperature, and then the number of viable bacteria colonizing the surface of these beetles was determined. These insects were selected due to their hardiness, availability, and low production of endogenous alarm compounds. Insects containing OCT and DEC on their surface exhibited significantly less bacterial colonization compared to those coated in DMSO (Figure 3). This indicates that in addition to acting as an anti-predatory alarm signal, trans-2-octenal and trans-2-decenal of the brown marmorated stink bug (*Halyomorpha halys*) exhibit antimicrobial effects which presumably limit bacterial colonization and infection.

Previous studies indicated that stink bug aldehydes exhibited antifungal activity [9]. This led us to hypothesize that these compounds may also inhibit bacterial growth. Therefore, we tested whether OCT and DEC, two of the most prominent stink bug alarm aldehydes released by the brown marmorated stink bug, exhibited antibacterial activity. Since stinkbugs can release microliter amounts of OCT and DEC daily, the initial concentration used here on the beetles would be slightly higher than that produced naturally, but would evaporate to concentrations found on the stinkbugs normally over the course of the experiment [15]. The *in vitro* and *in vivo* findings presented here suggest that stink bug aldehydes function to antagonize bacterial colonization and infection. These compounds may have potential for human use as antimicrobial agents. Future work should determine whether these compounds are toxic to humans, and if they maintain efficacy upon administration to a mammalian host during infection.

Earwigs have been shown to exude benzoquinones which not only repel predators, but also provide protection against microbes and nematodes. Future studies will focus on determining whether stink bug aldehydes likewise exhibit anti-nematode activity [10].

Because OCT and DEC inhibit bacterial growth, these compounds may have potential for application as antimicrobials. Aldehydes are reactive compounds that are capable of cross-linking biologically important molecules such as DNA and proteins; hence, this group of chemicals has been used for decades in antimicrobials such as disinfectants, preservatives, and antiseptics [16]. Therefore, based on the data presented here, the brown marmorated stink bug represents a plentiful, natural source of aldehydes having potential to be harvested for application as antimicrobial chemicals. However, based on the unfavorable odor of these compounds, future work should focus on chemically modifying OCT and DEC to remove the noxious properties, while maintaining antimicrobial activity.
